# COVID-19 Detection Through Transfer Learning Using Multimodal Imaging Data

**DOI:** 10.1109/ACCESS.2020.3016780

**Published:** 2020-08-14

**Authors:** Michael J. Horry, Subrata Chakraborty, Manoranjan Paul, Anwaar Ulhaq, Biswajeet Pradhan, Manas Saha, Nagesh Shukla

**Affiliations:** Centre for Advanced Modelling and Geospatial Information Systems (CAMGIS), School of Information, Systems, and Modeling, Faculty of Engineering and ITUniversity of Technology Sydney 1994 Sydney NSW 2007 Australia; Machine Vision and Digital Health (MaViDH), School of Computing and MathematicsCharles Sturt University 1109 Bathurst NSW 2795 Australia; Manning Rural Referral Hospital Taree NSW 2430 Australia; Department of Energy and Mineral Resources EngineeringSejong University 35006 Seoul 05006 South Korea; IBM Australia Limited Sydney NSW 2065 Australia

**Keywords:** COVID-19 detection, image processing, model comparison, CNN models, X-ray, ultrasound and CT based detection

## Abstract

Detecting COVID-19 early may help in devising an appropriate treatment plan and disease containment decisions. In this study, we demonstrate how transfer learning from deep learning models can be used to perform COVID-19 detection using images from three most commonly used medical imaging modes X-Ray, Ultrasound, and CT scan. The aim is to provide over-stressed medical professionals a second pair of eyes through intelligent deep learning image classification models. We identify a suitable *Convolutional Neural Network* (CNN) model through initial comparative study of several popular CNN models. We then optimize the selected VGG19 model for the image modalities to show how the models can be used for the highly scarce and challenging COVID-19 datasets. We highlight the challenges (including dataset size and quality) in utilizing current publicly available COVID-19 datasets for developing useful deep learning models and how it adversely impacts the trainability of complex models. We also propose an image pre-processing stage to create a trustworthy image dataset for developing and testing the deep learning models. The new approach is aimed to reduce unwanted noise from the images so that deep learning models can focus on detecting diseases with specific features from them. Our results indicate that Ultrasound images provide superior detection accuracy compared to X-Ray and CT scans. The experimental results highlight that with limited data, most of the deeper networks struggle to train well and provides less consistency over the three imaging modes we are using. The selected VGG19 model, which is then extensively tuned with appropriate parameters, performs in considerable levels of COVID-19 detection against pneumonia or normal for all three lung image modes with the precision of up to 86% for X-Ray, 100% for Ultrasound and 84% for CT scans.

## I. Introduction

The current COVID-19 pandemic has impacted the world with over 18.35 million infections and over 6,96,147 deaths so far (as of $5^{\text {th}}$ August 2020) [1]. Early identifying, isolation and care for patients is a key strategy for a better management of this pandemic. Our study aims to provide a conceptual transfer learning framework to support COVID-19 detection with the use of image classification using deep learning models for multiple imaging modes including X-Ray, Ultrasound, and CT scan. The acquisition of a sufficiently large, publicly available corpus of medical image sample data for fully training deep learning models is challenging for novel medical conditions such as COVID-19 since collection and labelling of images requires significant time and resources to compile. An alternative method of training deep learning models is “transfer learning” whereby a deep learning network is pre-weighted with the results of a previous training cycle from a different domain. This technique is commonly used as a basis for initializing deep learning models which are then fine-tuned using the limited available medical sample data set with results that have been documented to outperform fully trained networks under certain circumstances [2], [3]. The study will demonstrate how transfer learning can be used for COVID-19 detection for three commonly used imaging modes X-Ray, Ultrasound, and CT scan. This could assist practitioners and researchers in developing a supporting tool for highly constrained health professionals in determining the course of treatment. The study further demonstrates a pre-processing pipeline for improving the image quality, for deep learning-based predictions. An initial testing is also conducted to understand the suitability of various popular deep learning models for the limited available dataset in order to select a model for the proposed image classification demonstrations on multiple image modes.

Fast, accessible, affordable and reliable identification of COVID-19 pathology in an individual is key to slowing the transmission of COVID-19 infection. Currently, reverse transcriptase quantitative polymerase chain reaction (RT-qPCR) tests are the gold standard for diagnosing COVID-19 [4]. During this test small amounts of viral RNA are extracted from a nasal swab, amplified, and quantified with virus detection indicated visually using a fluorescent dye. Unfortunately, the RT-qPCR test is manual and time-consuming, with results taking up to two days. Some studies have also shown false positive Polymerase Chain Reaction PCR testing [5]. Other testing approaches include imaging technology-based approaches including computed tomography (CT) imaging [6] and X-Ray imaging based [7], [8] and Ultrasound imaging [9].

The CT scan-based COVID-19 detection is time consuming and manual with the requirement of expert involvements. CT scanning machines are also difficult to use for COVID patients, as the patients often need to be transferred to the CT room, the machines would require extensive cleaning after each usage, and higher radiation risks [10]. Although CT is not recommended as a primary diagnostic tool, it has been successfully used as a supporting tool for COVID-19 condition assessment [6]. Common CT findings include ground-glass opacities (GGO) at the early stage, during progressive stage, air space consolidation during peak stage, Broncho vascular thickening in the lesion, and traction bronchiectasis are visible during absorption stage [10]. Several studies have shown promising results in using deep learning models to automated diagnosis of COVID-19 from CT images [6], [11], [12]. Both the PCR tests and CT scans are comparatively costly [13], [14] and with an overwhelming demand many countries are forced to perform selective testing for only high-risk population.

X-Ray imaging is relatively cost effective and commonly utilized for lung infection detection and is useful for COVID-19 detection as well [15]. Medical observations were made by one of the co-authors of this research (Dr. Saha) who is also a medical professional, as well as by treating doctors of the COVID-19 dataset [16] patients. The common features observed in the X-Ray images of patients with COVID-19 are patchy infiltrates or opacities that bear similarities to other viral pneumonia features. X-Ray images do not show any abnormalities in the early stages of COVID-19. However, as the disease progresses, COVID-19 gradually manifests as a typical unilateral patchy infiltration involving mid zone and upper or lower zone of the lungs, occasionally with evidence of a consolidation.

Ultrasound imaging has also been recommended as a tool for COVID-19 lung condition assessment since it can be used at bedside with minimal infection spreading risks and has excellent ability to detect lung conditions related to COVID-19 [17]. Progression of COVID-19 infection is evident as B-lines aeration in early stages of consolidation in critical stages [10], [18].

**FIGURE 1.**
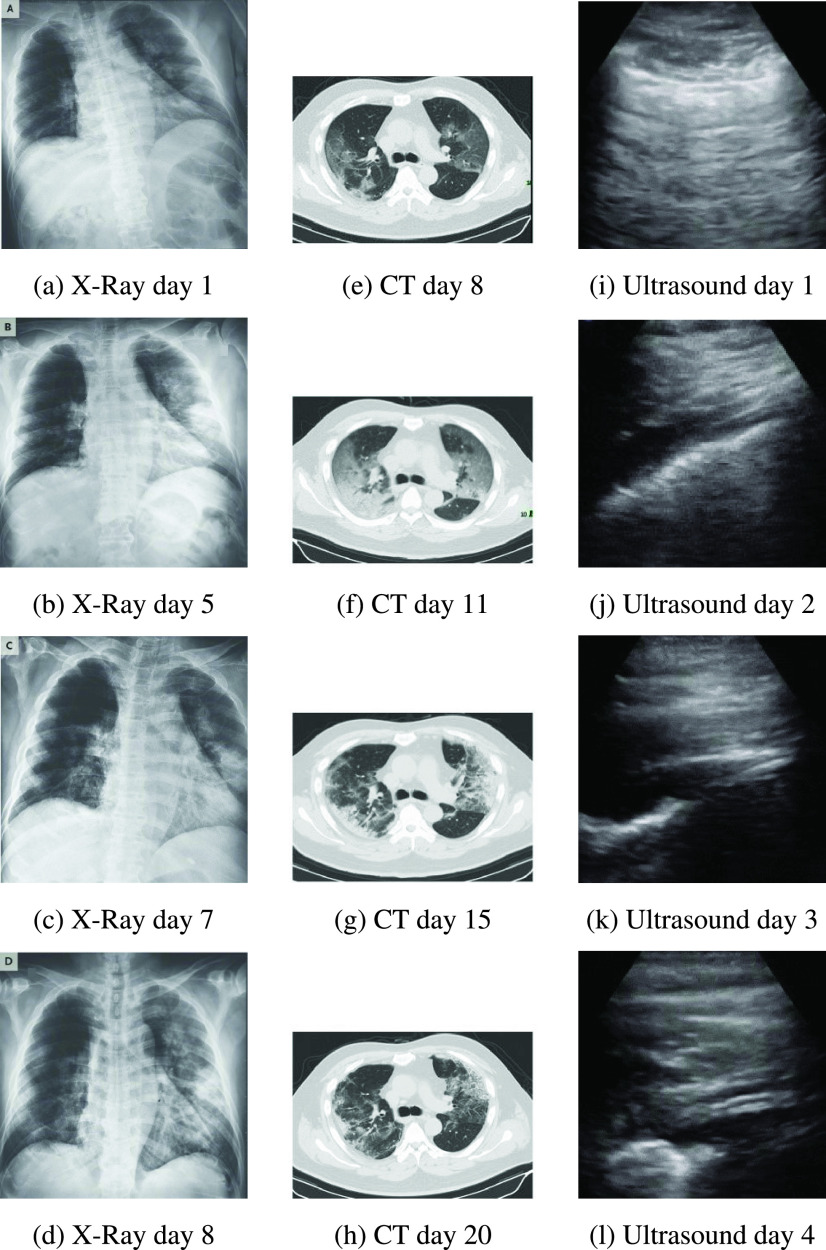
COVID-19 progression over several days as evident in different imaging modes.

Fig. 1 shows the progression of evidence for the patient in the COVID-19 datasets for X-Ray, CT and Ultrasound imaging.

Computer vision diagnostic tools for COVID-19 from multiple imaging modes such as X-Ray, Ultrasound, and CT would provide an automated “second reading” to clinicians, assisting in the diagnosis and criticality assessment of COVID-19 patients to assist in better decision making in the global fight against the disease. COVID-19 often results in pneumonia, and for radiologists and practitioners differentiating between the COVID-19 pneumonia and other types of pneumonia (viral and bacterial) solely based on diagnostic images could be challenging [19].

Deep learning artificial neural networks, and the Convolutional Neural Networks (CNNs) have proven to be highly effective in a vast range of medical image classification applications [20], [21]. In this study, we present three key contributions. Primarily, we demonstrate how transfer learning capabilities of off the shelf deep learning models can be utilized to perform classifications in two distinct scenarios for three imaging modes X-Ray, Ultrasound, and CT scan:

1) Identifying the pneumonia (both COVID-19 and other types) affected lung against the normal lung.
2) Identifying COVID-19 affected lung from non COVID-19 pneumonia affected lung.

Secondly, we present a comparative study in order to select a suitable deep learning model for our demonstration. We performed a comparative testing of several common off-the-shelf CNN models namely VGG16/VGG19 [22], Resnet50 [23], Inception V3 [24], Xception [25], InceptionResNet [26], DenseNet [27], and NASNetLarge [28]. The testing is not intended for exhaustive performance comparison of these methods, rather we wanted to select the most suitable one for our multi-modal image classification, which performs decently with minimal tuning. The source X-Ray, Ultrasound, and CT image samples, especially those from the COVID-19 data sets, have been harvested from multiple sources and are of inconsistent quality. In our final contribution, we have implemented a pre-processing pipeline to reduce unwanted signal noise such as non-lung area visible in X-Rays, and thereby reduce the impact of sampling bias on this comparison. Through this pre-processing pipeline, we minimize the image quality imbalances in the image samples. This would allow models to train on lung features only thus having a greater chance of learning disease features and ignoring other noise features. The study would provide timely model selection guidelines to the practitioners who often are resorted to utilise certain mode of imaging due to time and resource scarcity.

In the following sections we first present a brief review of recent scholarly works related to this study, followed by a discussion on the datasets we used and related challenges. We then present the dataset generation process along with our proposed pre-processing pipeline for data quality balancing. We then present the deep learning model selection process along with comparison results. Finally, we present the performance results with discussions for our selected model on all three image modes.

## II. Related Work

Computer aided detection and diagnosis of pulmonary pathologies from X-Ray images is a field of research that started in the 1960s and steadily progressed in the following decades with papers describing highly accurate diagnosis of a range of conditions including osteoporosis [29], breast cancer [30], and cardiac disease [31].

CT scans also use X-Rays as a radiation source, however, they provide much higher image resolution and contrast compared to standard X-Ray images because of a much more focused X-Ray beam used to produce cross-sectional images of the patient [32]. CT is generally considered as the best imaging modality for lung parenchyma and is widely accepted by clinicians as the “gold standard” [33]. A large corpus of research exists relating to the use of machine learning to improve the efficiency and accuracy of lung cancer diagnosis – largely driven by extensive CT based lung cancer screening programs in many parts of the world. Several researches have achieved incredibly accurate results using CNNs with transfer learning to detect lung nodules [34]–[37]. Recently a deep learning system built by Google achieved state-of-the-art performance using patients’ current and prior CT volumes to predict the risk of lung cancer. This system outperformed human radiologists where prior CT scans were not available, and equaled human radiologist performance where historical CT scans were available [38]. Although X-Ray is the current reference diagnosis for pneumonia, some studies point out that CT generally outperforms X-Ray as a diagnostic tool for pneumonia, albeit at higher cost and convenience [39], [40].

Ultrasound has traditionally been used diagnostically in the fields of cardiology and obstetrics and more recently for a range of other conditions covering most organs. One of the reasons for this increase in the use of ultrasound is that technical advancements including machine learning have allowed useful information to be determined from the low quality and high signal-to-noise images that are typical of the Ultrasound imaging modality [41]. Several researchers have recently used Ultrasound as an effective diagnostic aid in hepatic steatosis, adenomyosis, and craniosynostosis [42], Pancreatic cancer [43], Breast cancer [44] and prostate cancer [45]. Use of bedside ultrasound in critically ill patients compared favorably against chest X-Ray and approached the diagnostic accuracy of CT scans for a range of thoracic conditions [46]. The combination of lung ultrasound with machine learning techniques was found to be valuable in providing faster and more accurate bedside interpretation of lung ultrasound for acute respiratory failure [47].

Difficulties in distinguishing soft tissue caused by poor contrast in X-Ray images have led some researchers to implement contrast enhancement [48] as a pre-processing step in X-Ray based diagnosis. In addition, lung segmentation of X-Ray images is an important step in the identification of lung nodules and various segmentation approaches are proposed in the literature based on linear filtering/thresholding, rolling ball filters and more recently CNNs [49].

Although CT scans are much higher contrast/resolution compared to X-Ray factors such as low dose and improper use of image enhancement can lead to poor quality images. A number of researchers have noted that histogram equalization techniques, particularly adaptive histogram equalization can improve the contrast of CT images [50]. A combination of histogram normalization, gamma correction and contrast limited adaptive histogram equalization has been shown to objectively improve the quality of poor contrast CT images [51].

Ultrasound images tend to be noisy due to the relatively low penetration of soundwaves into organic tissue compared to X-Rays. This limitation has led a number of researchers to develop methods to improve the quality of ultrasound images by various means including noise filtering, wavelet transformation and deconvolution [52]. Contrast Limited Adaptive Histogram Equalization (CLAHE) has been used as part of a pre-processing pipeline to enhance the quality of ultrasound images [53].

In the literature review we noted a small number of very recent studies that have used deep learning systems for COVID-19 screening and diagnosis. A custom-built 18-layer residual network pre-trained on the ImageNet weights against COVID-19 (100 images) and Pneumonia (1431 images) X-Ray image datasets [54]. A range of deep learning frameworks coined as COVIDX-Net trained on a small data set of 25 confirmed COVID-19 cases [55]. A custom curated dataset of COVID-19, viral pneumonia and normal X-Ray images [56]. A custom residual CNN that was highly effective in distinguishing between COVID-19, Pneumonia and normal condition X-Ray images [57]. These studies used the COVID-19 dataset [16] for the COVID-19 X-Ray samples, and the RSNA dataset [58] was used to get pneumonia and normal X-Ray samples.

Automated COVID-19 Pneumonia diagnosis from CT scans has been the focus of recent studies with promising results [59]–[62]. A combined U-Net segmentation and 3D classification CNN has been used to accurately predict the presence of COVID-19 with an accuracy of 90% using a non-public dataset of CT images [63]. A ResNet50 based CNN with transfer learning from the ImageNet weights was able to classify COVID-19 with 94% accuracy [64] against a normal condition CT slice using unspecified multiple international datasets as a corpus. In a recent work, [65] addressed the challenge of automatically distinguishing between COVID-19 and community acquired pneumonia using machine learning. This system uses a U-Net pre-processor model for lung field segmentation, followed by a 3D ResNet50 model using transferred ImageNet weights. This study achieved a sensitivity of 87% against a large non-public dataset collected from 6 hospitals. The DenseNet-169 CNN has been used [66] to detect COVID-19 vs non-COVID-19 CT slices. Without segmentation this system achieved an accuracy of 79.5% with an F1 score of 76%. Using joint segmentation, the classification accuracy was raised to 83.3% with an F1 score of 84.6%.

There has been less attention given to the use of machine learning to automate COVID-19 diagnosis from Ultrasound images, however a ResNet based CNN trained on the available Ultrasound COVID-19 data has achieved an accuracy of 89% with recall accuracy for COVID-19 of 96% [9].

Each imaging mode differs in terms of cost/availability and the level of clinical expertise required to accurately interpret the generated medical images. Different imaging modes are therefore suitable to different contexts – for example both X-Ray and Ultrasound can be implemented as low-cost portable units that may be used as bedside or even as field diagnostic tools. CT scanning equipment is typically physically fixed at high cost and is therefore only available within the confines of hospitals and medical clinics. Our main aim is to first select one suitable deep learning model through comparative testing of a range of off-the-shelf deep learning models against each of these imaging modes using transfer learning. The comparison results are then used to address limited sample data size and data variability. We then applied image pre-preprocessing to improve image quality and reduce inter and intra dataset systematic differences in brightness and contrast level. Finally, we performed extensive parameter tuning on the selected model and compared the performance of this model for each imaging mode.

## III. Dataset Development

### A. Data Sourcing

Large numbers of X-Ray, CT and Ultrasound images are available from several publicly accessible datasets. With the emergence of COVID-19 being very recent none of these large repositories contain any COVID-19 labelled data, thereby requiring that we rely upon multiple datasets for Normal, Pneumonia, COVID-19 and other non COVID-19 source images.

COVID-19 chest X-Rays were obtained from the publicly accessible COVID-19 Image Data Collection [16]. This collection has been sourced from websites and pdf format publications. Unsurprisingly, the images from this collection are of variable size and quality. Image contrast levels, brightness and subject positioning are all highly variable within this dataset. Our analysis in this article is based on a download of this dataset made on 11 May 2020.

The selection of a dataset for Normal and Pneumonia condition X-Rays posed a dilemma since a highly curated data set is not comparable to the available COVID-19 chest X-Ray dataset. Our early tests against one such dataset gave an unrealistically high classification accuracy for the quality of the data under test. We found that the National Institute of Health (NIH) Chest X-Ray [67] dataset – provided images are of a similar size, [68] quality and aspect ratio to the typical images in the COVID-19 dataset with dimensions being uniformly $1024 \times 1024$ pixels in a portrait orientation.

CT scans for COVID-19 and non COVID-19 were obtained from the publicly accessible COVID-CT Dataset [66]. This dataset has been sourced by extracting CT slice images showing the COVID-19 pathology from preprint papers. Once again, the images from this collection are of variable size and quality. Moreover, the process of CT scanning is dynamic, with a full scan consisting of many discrete slices taken in a helical pattern along the thoracic cavity. The images in this collection only present a single, or small number of slices per patient. As CT slice images progress along the thoracic cavity, the structural features visible in the generated image change dramatically. Ideally all slices would be available for analysis in order to equalize the distribution/prominence in the image of these features, however this is not the case with this dataset. Our analysis in this article is based on a download of this dataset made on 11 May 2020.

Ultrasound images for COVID-19, Pneumonia and Normal conditions were obtained from the publicly accessible POCOVID-Net data set [9]. These images have been sampled from video sourced from various online sources. We noticed a huge variation in the quality in the images within each condition caused by the position of the ultrasound apparatus on the patients’ chest. Ideally ultrasound video would be taken in a systematic way to allow for greater comparability of the condition datasets with every frame in the video subject to analysis. Neither of these conditions are satisfied by this dataset. Our analysis in this article is based on a download of this dataset made on 11 May 2020.

**FIGURE 2.**
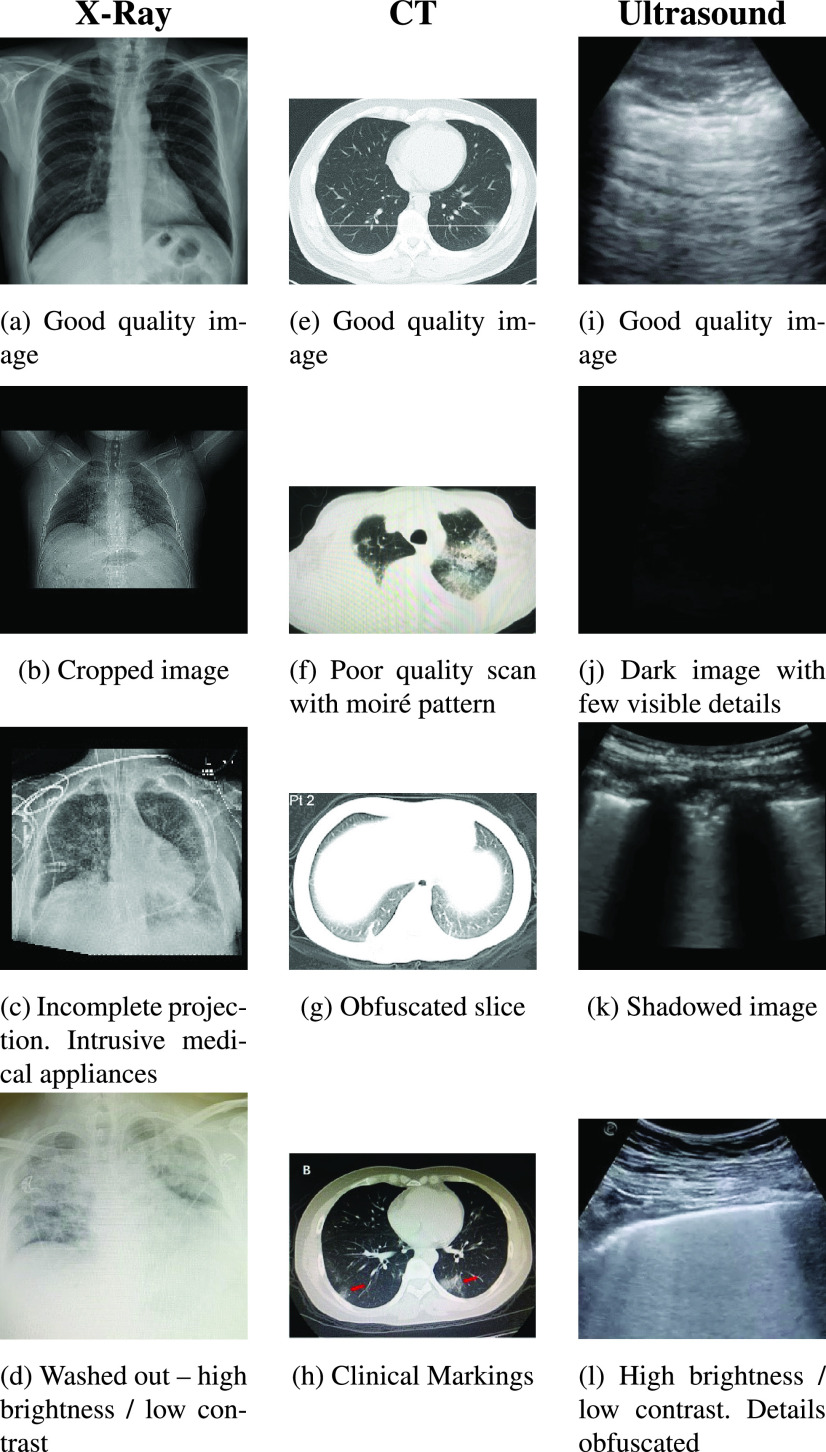
Different variations observed in the COVID-19 datasets.

The number of images of each dataset along with a description of the characteristics of the datasets is described in Table 1. We believe the significant quality variations between data from different classes need to be balanced for deep learning models to learn actual disease related variations. Therefore, our study stresses the importance of sampling bias/signal noise removal from the image datasets prior to using them for model development and classification in order to obtain meaningful and trustworthy classification results. Some illustrative examples of this variability of these datasets is shown in Fig. 2. Of these examples images (b), (c) and (f) appear to have been cropped from journal articles and in the case of (f) scanned. These images are of poor quality and lacking detail that would indicate a pathology to our machine learning models. Images (g), (j) and (k) also lack detail as a result of apparatus positioning. Images (d) and (l) show high brightness and low contrast, thus hiding pathological details. Despite the variability of the datasets we chose to only very lightly curate data as described in Section III(C) Data Pre-processing and shown in Table 2. Our reasoning for this is twofold. Firstly we wish to avoid biasing the data corpus with a non-expert subjective opinion of pathological indications, and secondly we consider the usefulness of this study to potentially extend to future pandemic situations where similar data quality issues will be likely if not inevitable.

**TABLE 1:** Summary of Data Sources Used

Collection	Number of Images	Characteristics	Notes
COVID-19 Image Data Collection [16]	115: COVID-19 (PA)	Variable size, quality, contrast and brightness.	Only source of publicly accessible COVID-19 PA X-Ray images and used in this study.
NIH Chest X-Ray [67]	322: Pneumonia 60361: No Finding	Intra-dataset uniformity similar to COVID-19 dataset. All images are 1024 x1024 in size.	Objectively similar in quality to the COVID-19 Image Data Collection. Used in this study.
COVID-CT Dataset [66]	349: COVID-19 397: Non COVID	Variable size, contrast and brightness	Only source of publicly accessible COVID-19 CT images and used in this study.
POCOVID-Net Dataset [9]	654: COVID-19 277: Pneumonia 172: No Finding	Variable size, contrast and brightness	Only source of publicly accessible COVID-19 Ultrasound images and used in this study.

**TABLE 2:** Sampled Dataset for Experiments

Image Mode	Condition	Source images	Curated images	Number of augmented images	Source
X-Ray	COVID-19	140	139	2,920	[16]
	Pneumonia	322	190	2,920	[67]
	Normal	60361	400	5,840	[67]
CT	COVID-19	349	349	6,000	[66]
	Non COVID	397	397	6,000	[66]
Ultrasound	COVID-19	399	399	2,720	[9]
	Pneumonia	277	277	2,720	[9]
	Normal	235	235	5,440	[9]

### B. Data Sampling

In this study, we aim to use real X-Ray, Ultrasound and CT scan data only, and not considering creation and use of synthetic data at this stage. We also used a relatively balanced dataset size for our model experiments, with imbalance addressed using calculated class training weights. From the source datasets shown in Table 1, we created a master dataset for our experiments.

The X-Ray COVID-19 dataset was lightly curated to remove a single image that was an incorrectly labelled projection. All other COVID-19 images were included for the various modes. The non-COVID-19 images were also lightly curated to remove images that were mislabeled projections or dominated by intrusive medical devices. This left us with usable image samples for X-Ray, Ultrasound and CT scan to work with. Since the resulting sample corpus was still relatively small for a deep learning application, we applied several data augmentation transformations including horizontal flip, horizontal and vertical shift and rotation during the experiment process to increase the volume and variety of the sample set as summarized in Table 2.

We recognized that although we were using only a small number of image collections for our experiments; we were in fact relying upon data sourced from an unknown number and variety of X-Ray, CT, and Ultrasound machines, each with variable exposure parameters and operator behaviors. Systematic image exposure and brightness differences within and between the datasets of each imaging mode proved to be particularly concerning, and several researchers have indicated that medical image analysis methods are highly sensitive to variations of image intensities [69]. Research has shown that the feasibility of an automated analysis system requires that “the intensity of the same tissue should always be similar in one image and not vary with the location of the tissue” [69]. This principle, when extrapolated to the many images utilized in machine learning algorithms, implies that all images in the sample data sets should have similar intensity for the same tissue over the entire set of images as well as within a single image.

Since the machine learning classifiers, use a pixel array as a data source, any systematic difference in pixel intensity between the datasets would introduce sampling bias in the results. This would have the consequence of training the machine learning classifiers on systematic image histogram differences rather than the actual clinical image content of interest.

To minimize the effect of sampling bias, we applied histogram equalization to images using the N-CLAHE method described by [70]. The method both normalizes images and enhances small details, textures and local contrast by first globally normalizing the image histogram followed by application of Contrast Limited Adaptive Histogram Equalization (CLAHE) [71]. This was implemented using the OpenCV equalizeHist and createCLAHE functions [72].

**FIGURE 3.**
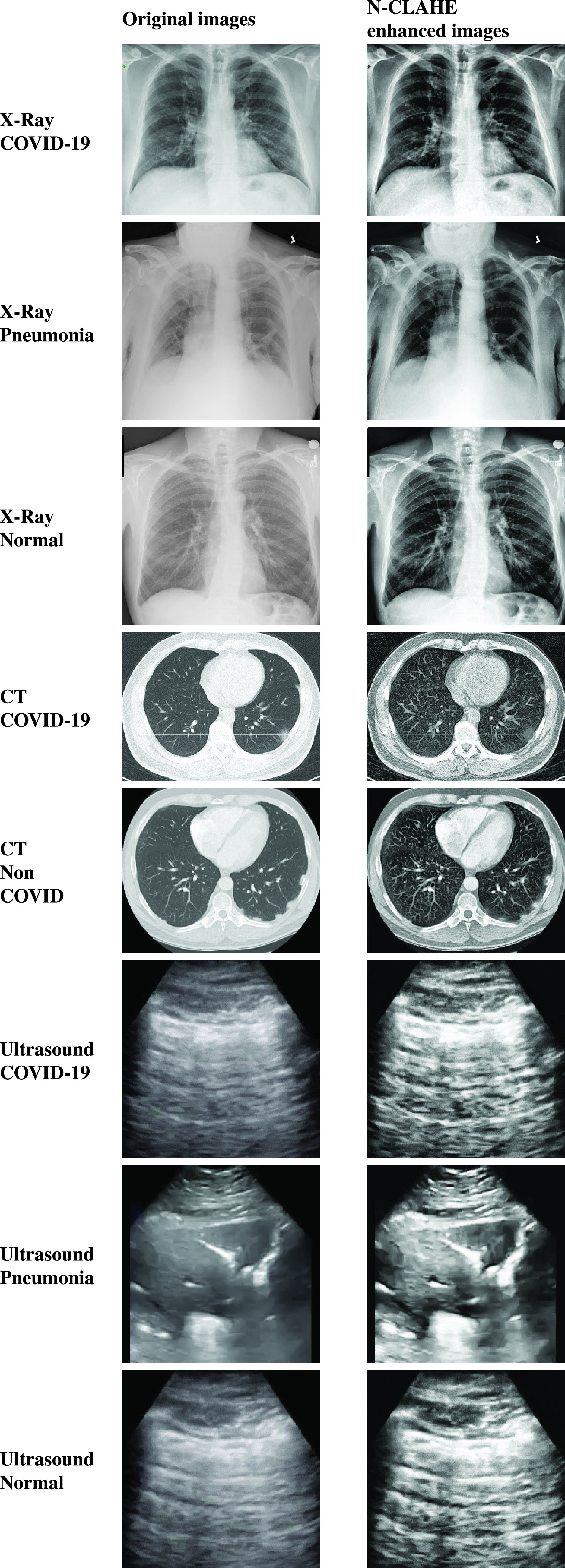
Results of enhancement preprocessing on original samples for COVID-19, Pneumonia and Normal images.

As shown in Fig. 3, the N-CLAHE pre-process greatly improved the image brightness/contrast consistency across the datasets as well as enhancing the fine details of these images. The same effect was observed within each data set. Subjectively, the authors can no longer easily tell which image has been drawn from which dataset purely on image brightness and contrast characteristics alone.

## IV. Model Development

### A. Classification Pipeline

**FIGURE 4.**
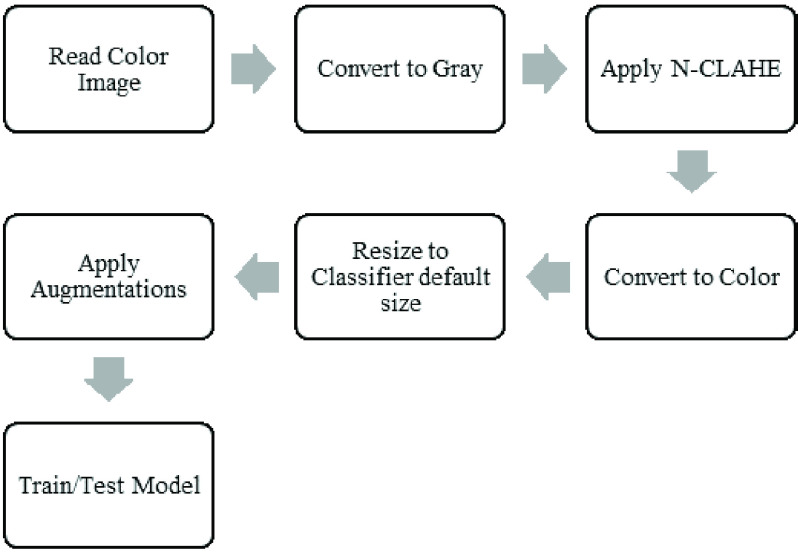
Experiment pipeline for preprocessing and classification.

The experiment pipeline is shown in Fig. 4. Unprocessed images are read with directory names used as class labels. N-CLAHE is then applied to normalize images and highlight the finer details for the attention of the machine learning classifiers. Images are then resized to the classifier default size, for example $224\times 224$ pixels for VGG16/19 and $299\times 299$ pixels for InceptionV3. Following image resizing, data augmentation is applied to increase the number and variation of images provided to the classifier. Augmentations applied include horizontal flip, rotation, width shift, and height shift. Vertical flip was not applied since X-Ray images are not vertically symmetrical, and the resulting flipped image would not resemble a real chest X-Ray. Finally, the augmented images are utilized by the machine learning classifier using an 80:20 Train/Test split.

### B. Model Consideration

One of the key objectives of this study was to achieve reliable classification results using publicly accessible data and “out-of-the-box” models with transfer learning to both compensate for the limited size of the sample data sets, and to accelerate the training process so that this could be reasonably performed on modest hardware.

CNN based models are well utilized for image classification purposes and we want to initially select a suitable CNN based deep learning model for our multimodal image classification study. Our primary aim is not to perform exhaustive performance evaluation among all available models, rather we aim to show the generic applicability of popular model genres for the challenging and limited time critical dataset for COVID-19 chest images in multiple modes including X-Ray, CT and Ultrasound to provide reasonable precision.

Since the ImageNet challenge (ILSVRC) 2012 [73], there has been a flurry of development in deep learning models for image classification and computer vision applications. These developed models can be broadly grouped into several distinct model families such as AlexNet, VGG Nets, Inception Nets, ResNets, MobileNets, DenseNets and NASNets based on their distinct architectures [74], [75]. Over the years, these basic model families produced several versions [74] and they have been extensively used by other researchers to develop modified and hybrid models [76]. Recent studies attempted to improve performance of the base models by proposing new layers and filters such as Sparse Shift Filter [77], Asymmetric Convolution Block [78], Adder Networks [79], Virtual Pooling [80], Discrete Wavelet Transform [81], and HetConv [82], etc. Some recent substantial models have been developed based on the base models, such as Res2Net [83] and Wide ResNet [84] using the ResNet model; while Log Dense Net [85] and Sparse Net [86] using the DenseNet model. Another path of development combining multiple base models has resulted in a number of hybrid models including AOGNet [87], PNASNet [88], AmoebaNet [89], DPN [90], HCGNet [76], GCNet [91], ThiNet [92], and SKNet [93] etc.

In order to find the most suitable model for our study, we focused on widely popular models, suitable for transfer learning, and readily available in packaged form through trusted public libraries such as Keras. Hence, we only considered representatives of the base models in this domain as discussed below. Conveniently, these models are all available as part of the Keras API and each support transfer learning [94] in the form of supporting the pre-application to the model of the ImageNet [95] weights.

#### 1) VGG16 and VGG19

VGG16 and VGG19 [22] are convolutional neural network (CNN) architectures with very small convolution filters ( $3\times 3$) and a stride of 1 designed to achieve high accuracy in large-scale image recognition applications. The two implementations differ in depth of convolution/max-pooling and fully connected layers, with VGG16 having 16 layers in the base model and VGG19 having 19 layers.

#### 2) ResNet50 V2

The ResNet [23] CNN was developed as a means of avoiding the vanishing gradient problem inherent in deep neural networks by implementing a system of skip connections between layers – known as residual learning. This architecture results in a network that is more efficient to train, allowing for deeper networks to be designed that positively impact the model accuracy. ResNet50 is such a network with 50 layers of implementing residual learning.

#### 3) Inception V3

The Inception V3 [24] CNN aimed to improve utilization of computing resources inside the network by increasing the depth and width of the network whilst keeping computation operations constant. The designers of this network coined the term “inception modules” to describe an optimized network structure with skipped connections that is used as a building block. This inception module is repeated spatially by stacking with occasional max-pooling layers to reduce dimensionality to a manageable level for computation.

#### 4) Xception

The Xception [25] CNN was developed by Google Inc. as an “extreme” version of the Inception model. The Inception modules described above are replaced with depth wise separable convolutions. This Xception was shown to outperform Inception on a large-scale image classification dataset (comprising 350 million images of 17,000 classes).

#### 5) Inceptionresnet V2

The InceptionResNetV2 CNN [26] combines the Inception and Resnet architecture with the objective of achieving high classification performance using a ResNet architecture with the low computation cost of the Inception architecture.

#### 6) NASNetLarge

The NASNet (Large) CNN [28] has been developed by Google Brain as a data driven dynamic network that uses reinforcement learning to determine an optimal network structure for the image classification task at hand.

#### 7) DenseNet121

The DenseNet 121 CNN [27] uses shorter connections between layers in order to allow more accurate and efficient training on very deep networks.

### C. Model Selection

The Models discussed in the earlier section was firstly tuned using Keras-tune to determine an optimum range of learning rate, hidden network size and dropout rate. From this process, optimal hyperparameter ranges were determined to be:

• Learning Rate = 10^−3^ – 10^−5^,
• Hidden Layer Size = 8 – 96 neurons,
• Dropout =0.1 – 0.2

Each model was then trained 5 times over 100 epochs with precision, recall, training/testing accuracy and loss metrics captured along with training curves and confusion matrices for further analysis.

The test was repeated for learning rates between 10^−3^ and 10^−5^ with order-of-magnitude increments. The hidden layer size was also varied between 8 and 96. The batch size was varied between 2 and 16. Each classifier was trained on the ImageNet [95] weights for transfer learning. Finally, where models converged well, the best training hyperparameters were selected by inspection of training curves. The number of training epochs was then adjusted to prevent overfitting. The training and testing were then repeated with selected epochs and optimized hyperparameters to obtain performance scores.

The testing results as shown in Table 3 that the simpler VGG classifiers were more trainable on all three image modes and provided more consistent results across all three image modes. We also noted that ultrasound provided best classification results across all deep learning models compared to the CT and X-Ray image modes. The more complex models tended to either overfit in early epochs (<10) or failed to converge at all. Where reasonable results were obtained from the more complex models training curves typically showed overfitting and somewhat erratic training behavior in several cases. We also found that the more complex model trainability was highly dependent upon initial model hyperparameter choice, whereas the VGG classifiers produce good results for a wider range of hyperparameter choices.

**TABLE 3:** Model Performance Summary

Model	Image mode	Training Notes	Testing F1 (avg)
VGG16	X-Ray	Converged well. Overfitting evident at 30 epochs.	0.79
	Ultrasound	Converged well. Overfitting not evident at 100 epochs.	0.99
	CT	Converged. Overfitting evident from 5 epochs.	0.79
VGG19	X-Ray	Converged well. Overfitting not evident at 100 epochs.	0.87
	Ultrasound	Converged well. Overfitting not evident at 100 epochs.	0.99
	CT	Converged. Overfitting evident after 20 epochs.	0.78
Xception	X-Ray	Converged poorly. Overfitting evident after 10 epochs	0.76
	Ultrasound	Converged. Overfitting evident after 20 epochs.	0.82
	CT	Did not converge. Overfitting evident immediately	0.70
InceptionResNet	X-Ray	Converged poorly. Overfitting evident after 10 epochs.	0.73
	Ultrasound	Converged. Overfitting evident after 5 epochs.	0.89
	CT	Did not converge. Overfitting evident after 1st epoch.	0.63
InceptionV3	X-Ray	Converged poorly. Overfitting evident after 10 epochs.	0.75
	Ultrasound	Converged. Overfitting evident after 10 epochs.	0.87
	CT	Did not converge. Overfitting evident after 2 epochs.	0.71
NASNetLarge	X-Ray	Did not converge. Overfitting evident after 10 epochs.	0.64
	Ultrasound	Converged. Overfitting evident after 10 epochs.	0.80
	CT	Did not converge. Overfitting evident immediately.	0.64
DenseNet121	X-Ray	Converged poorly. Overfitting evident after 10 epochs.	0.74
	Ultrasound	Converged poorly. Overfitting evident immediately.	0.66
	CT	Converged. Overfitting evident after 8 epochs.	0.75
ResNet50V2	X-Ray	Converged. Overfitting evident after 20 epochs.	0.75
	Ultrasound	Converged well. Overfitting evident immediately.	0.93
	CT	Converged poorly. Overfitting evident immediately.	0.66

Finally, we noticed that the more complex models exhibited a higher training metrics deviation between epochs with randomly selected train/test splits. We believe the smaller data size and high fine-grained variability within the datasets were detected by the sensitive complex models, thus resulting in poorer performances. We expect complex model performance to improve with larger and better-quality data.

Based on our initial testing results, we have chosen the VGG19 model for our multimodal image classification testing in this study. We anticipate that future novel pandemics can also be expected to initially produce small, low quality medical image datasets and suggest that our findings are likely to extend to similar future applications with such challenging datasets.

The three modes of data (X-Ray, CT and Ultrasound) mainly used to understand lung conditions for a covid-19 patient. Through popular deep learning models, we try to understand their individual strength and weakness to detect COVID-19 lung condition for reasonable precision performance. This is vital for a doctor for decision making as each has advantages and disadvantages. Moreover, when there is limited time, resource, and patient condition, the doctor may need to take decision based on one modality. These results would help practitioners in selecting appropriate models for different imaging modes thus providing critical support when time and resources are stretched in a pandemic situation like the current COVID19.

## V. Experiments With Selected VGG19 Model

### A. Computing Equipment

All the experiments were performed on the University of Technology Sydney Interactive HPC environment under an Anaconda 3 software environment. Experiments were programmed using the Keras APIs with a TensorFlow 2 backend.

The server used was specified as an Intel Xeon Gold 6150 2.7GHz 18 cores (16 cores enabled) with 24.75MB L3 Cache (Max Turbo Freq. 3.7GHz, Min 3.4GHz). The server had 360GB RAM (Six Channel). This server hosted a NVIDIA Quadro P5000 GPU (2,560 Cores, 16GB Memory).

### B. Experiment Setup

**FIGURE 5.**
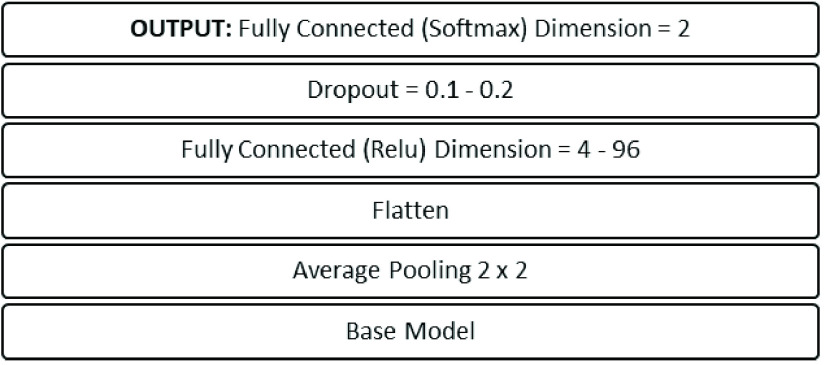
Head architecture of the proposed models.

VGG19 model was tuned for each image mode and each individual experiment to achieve the best possible results for the collated datasets. Learning rates were varied between 10^−3^ to 10^−6^ with order-of-magnitude increments. The batch sizes between 2 to 16 were applied. The hidden layer was varied between 4 and 96 nodes. Dropout rates of 0.1 and 0.2 were applied. These hyperparameter ranges generated an output head architecture as shown in Fig. 5.

### C. Experiment Dataset

The master dataset was utilized for training and testing with the VGG19 classifier over 5 experiments as shown in Table 4.

**TABLE 4:** Datasets Used for Experiments

Exp ID	Image Mode	Experiment	Dataset
1A	X-Ray	Normal vs (COVID-19 and Pneumonia)	(400 $\times$ Normal) vs (190 $\times$ Pneumonia 139 $\times$ COVID-19)
1B	Ultrasound	Normal vs (COVID-19 and Pneumonia)	226 $\times$ Normal vs (235 $\times$ COVID-19 220 $\times$ Pneumonia)
2A	X-Ray	COVID-19 vs Pneumonia	139 $\times$ COVID-19 vs 190 $\times$ Pneumonia
2B	Ultrasound	COVID-19 vs Pneumonia	235 $\times$ COVID-19 vs 220 $\times$ Pneumonia
3A	CT	COVID-19 vs Non COVID	349 $\times$ COVID-19 vs 397 $\times$ Non COVID

Processed dataset is available at: https://github.com/mhorry/N-CLAHE-MEDICAL-IMAGES

### D. Results and Discussions

**FIGURE 6.**
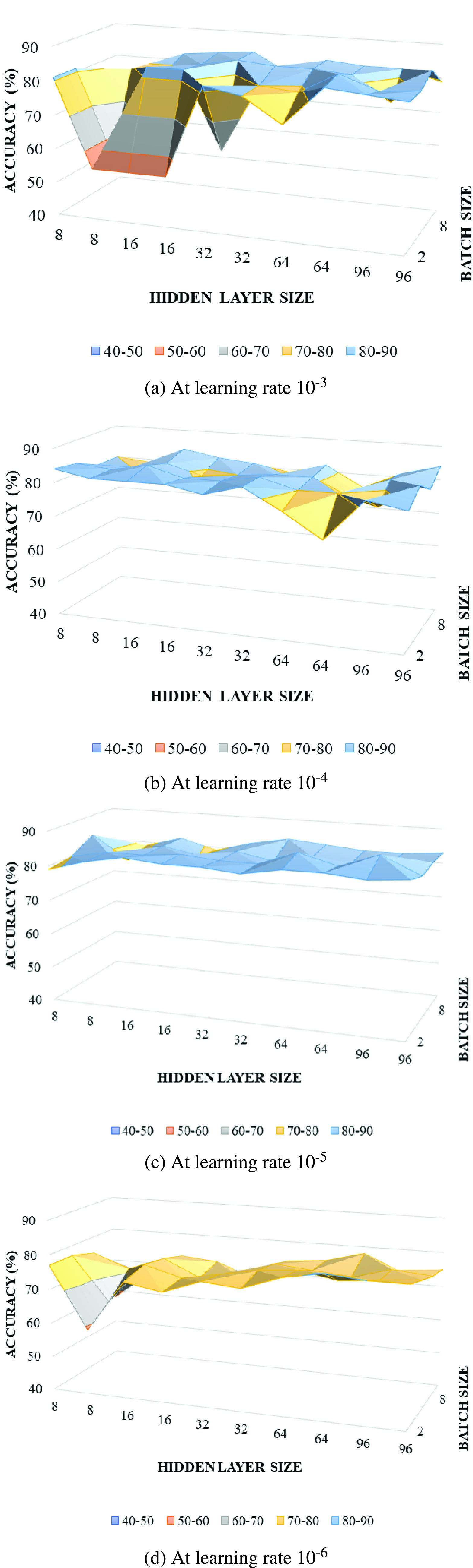
Model sensitivity to hyperparameters for experiment 1A.

With the selected VGG19 model for each experiment listed in Table 4, we first conducted the extensive performance tuning by adjusting multiple parameters including learning rate, batch size, node size and drop rate. The effects of learning rate, batch size and hidden layer size hyperparameter selection on the accuracy metric of experiment 1A is shown in Fig. 6. We noted that dropout rate had only a minimal effect on the model accuracy except at the highest learning rate of 10^−3^ and lowest learning rate of 10^−6^ where a dropout rate of 0.2 proved to be more stable than a dropout rate of 0.1. For learning rates for 10^−3^ and 10^−4^ the dropout rate selection has no discernable effect on model accuracy.

Learning rate, batch size and hidden layer size all affected model accuracy. The first observation is that learning rates of 10^−4^ and 10^−5^ provided higher model accuracy with 10^−5^ achieving more consistent results. There is a tendency for accuracy to improve with batch size increase at learning rates of 10^−3^ and 10^−6^ but at learning rates of 10^−4^ and 10^−5^ this tendency is not apparent. Finally, there is also a trend towards higher accuracy with a larger hidden layer size that is most noticeable at 10^−3^. Taking a learning rate of 10^−5^ as achieving consistent high accuracy, we can then suggest from this analysis that a hidden layer size ranging from 64 to 96 and batch size of 4 could generally be expected to provide the most accurate results for this experiment. Through similar analysis for each experiment in Table 4, we have identified the best parameter settings for each experiment as shown in Table 5.

**TABLE 5:** Experiment Results for Three Image Modes

Image mode	Experiment	Parameters	Classification	Results
X-Ray	1A	LR: 10^−5^ DR: 0.1 BS: 4 HS: 64 Epochs: 100	COVID-19 + Pneumonia	P: 0.85 R: 0.83 F1: 0.84
			Normal	P: 0.86 R: 0.88 F1: 0.87
Ultrasound	2A	LR: 10^−5^ DR: 0.2 BS: 2 HS: 64 Epochs: 100	COVID-19 + Pneumonia	P: 0.99 R: 0.97 F1: 0.98
			Normal	P: 0.94 R: 0.98 F1: 0.96
X-Ray	1B	LR: 10^−3^ DR: 0.2 BS: 8 HS: 8 Epochs: 100	COVID-19	P: 0.86 R: 0.86 F1: 0.86
			Pneumonia	P: 0.89 R: 0.89 F1: 0.89
Ultrasound	2B	LR: 10^−5^ DR: 0.2 BS: 2 HS: 64 Epochs: 100	COVID-19	P: 1.00 R: 1.00 F1: 1.00
			Pneumonia	P: 1.00 R: 1.00 F1: 1,00
CT	3A	LR: 10^−3^ DR: 0.2 BS: 4 HS: 16 Epochs: 70	COVID-19	P: 0.79 R: 0.83 F1: 0.81
			Non COVID	P: 0.84 R: 0.81 F1: 0.83

The results of the five experiments are listed in Table 5. For experiments classifying COVID-19 and Pneumonia vs Normal (1A and 2A) we found that the Ultrasound mode provided the best results with a sensitivity of 97% and positive predictive value of 99% compared to X-Ray with 83% and 85% respectively. For experiments classifying COVID-19 vs Pneumonia (1B and 2B) we again found that the Ultrasound mode provided the best results with a sensitivity of 100% and a positive predictive value of 100% compared to X-Ray with sensitivity of 86% and positive predictive value of 86%. The CT imaging mode was found to have a sensitivity of 83% and positive predictive value of 79% in classifying COVID-19 vs non COVID-19 scans. All experiments resulted in F1 scores exceeding 80% which is a good result given the relatively small and variable quality data corpus available.

**FIGURE 7.**
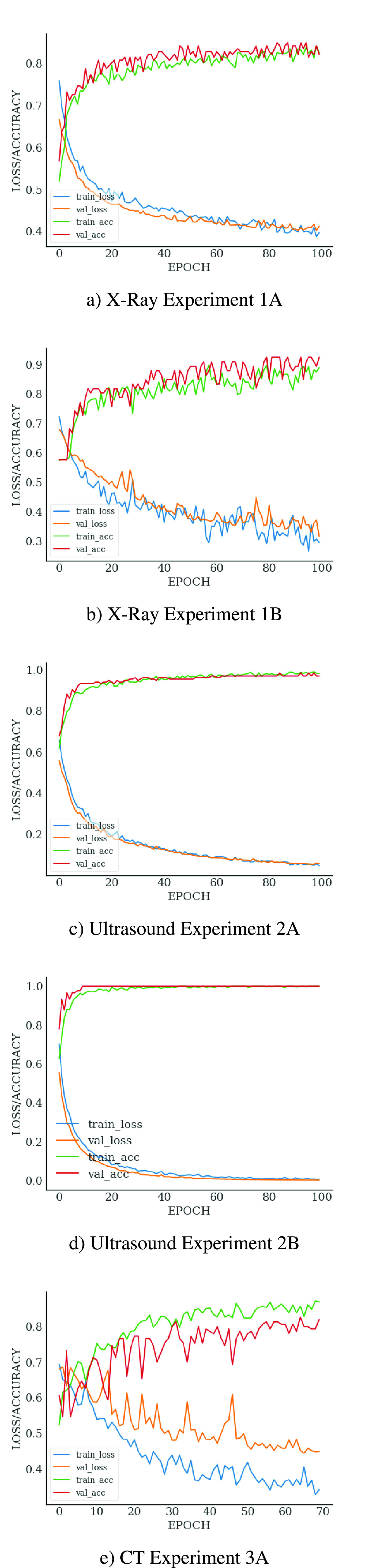
Learning curves for different modes.

The learning curves for each experiment are shown in Fig. 7. The training curves for both Ultrasound experiments (2A and 2B) are close to ideal. The training curves for the X-Ray experiments (1A and 2A) are also very good, although the curve for experiment 1B does show some signs of erratic learning patterns, which is the expected result of the highly variable image quality in the COVID-19 data set. The learning curve for the CT mode experiment (3A) is very erratic – even though the model did train, overfitting is arguably apparent after the epoch 50. Once again this is the expected result considering significant variation in the CT image data sets.

**FIGURE 8.**
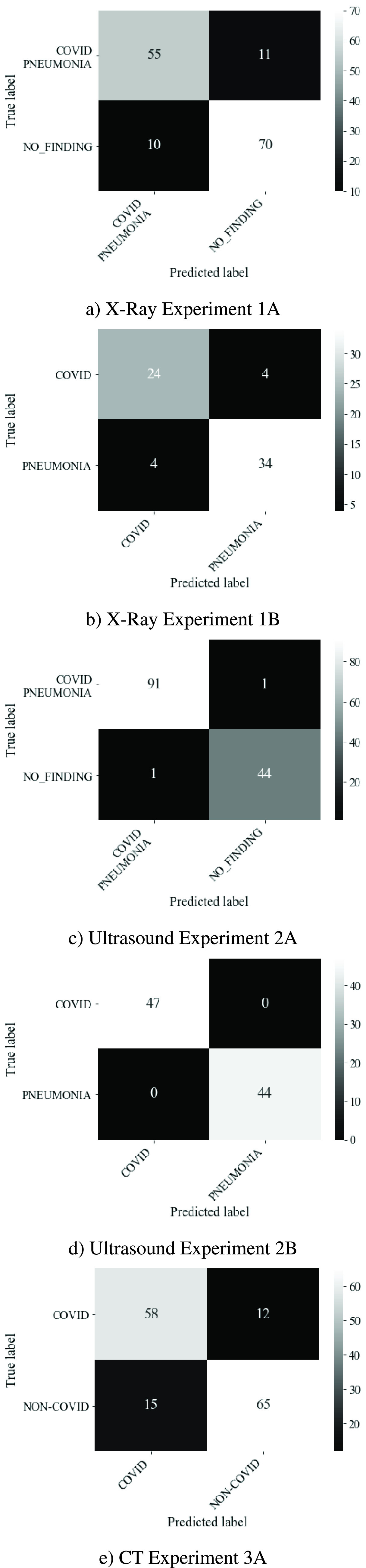
Confusion matrix for different modes.

The confusion matrices in Fig. 8 provides an indication of the false-negative and false-positive results of our experiments. Minimization of false negative predictions is important in the medical context since false reassurance may lead to diagnostic and treatment delay resulting in poor medical outcomes, patient mental distress, community loss in confidence relating to medical services and legal consequences [96]. False negative predictions for the Ultrasound mode experiments were very low at 1 and 0 for experiments 2A and 2B, respectively. False negative predictions for the X-Ray mode experiments were higher with 11 and 4 for experiments 1A and 1B respectively. The CT mode (experiment 3A) also performed poorly in this respect with 12 false negatives. Once again, the limited sample size and variable quality of the COVID-19 data sets used for the X-Ray and CT experiments are the most likely cause of the relatively high number of false negatives for experiments 1A, 1B and 3A.

**FIGURE 9.**
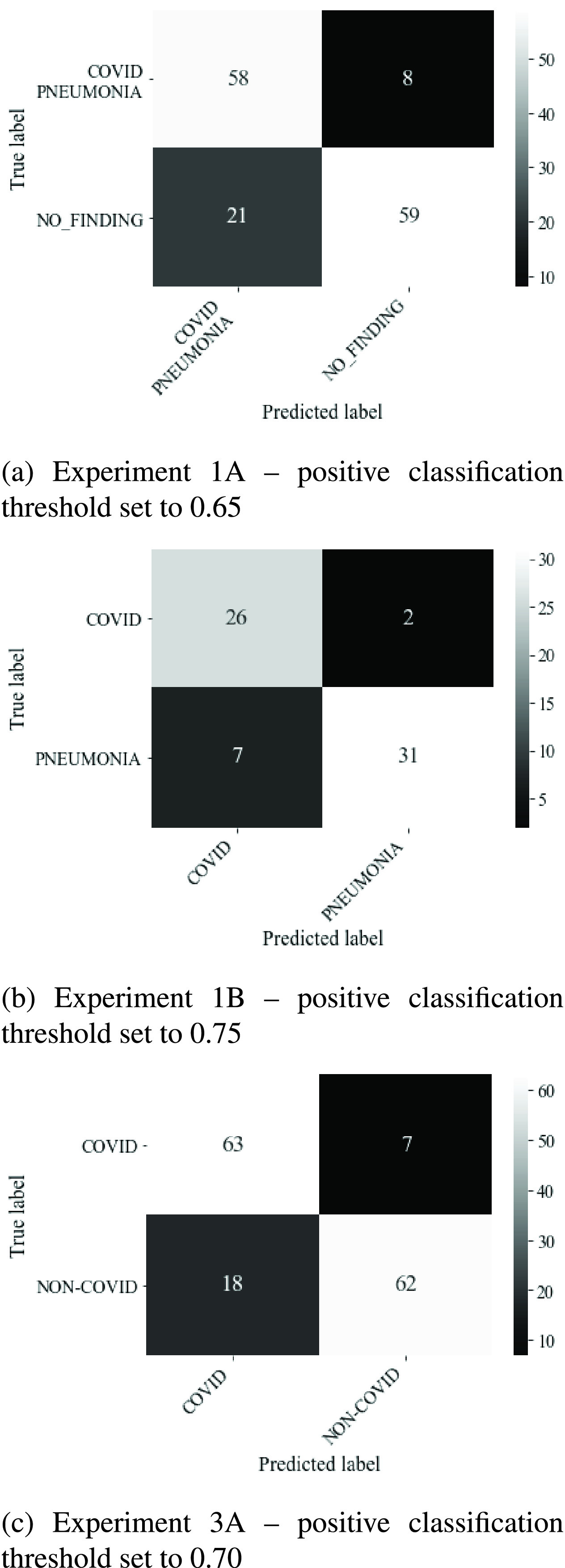
Reduction of false negatives using thresholding.

As previously noted, false negatives generated by the Keras class prediction threshold of 0.5 were high in the case of the CT and X-Ray imaging modes. We then performed adjustments in the class prediction threshold in 5% increments from 0.5 to 0.75. We successfully reduced false negatives, at the cost of increasing false positives. For experiment 1A we reduced false negatives from 11 to 8 using a threshold of 0.65. This raised the sensitivity of this test from 83% to 85% with a loss in positive predictive value from 85% to 73%. For experiment 1B we reduced false negatives from 4 to 2 using a threshold of 0.75. This raised the sensitivity of this test from 86% to 93% whilst reducing positive predictive value from 86% to 79%. For experiment 3A we reduced false negatives from 12 to 7 using a threshold of 0.7. This raised the sensitivity of this test from 83% to 90% with a reduction in positive predictive value from 79% to 78%. The confusion matrices associated with these results are shown in Fig. 9.

Overall, the results show that deep learning models were performing extremely well with the ultrasound image mode. The X-Ray and CT modes are challenging with the available COVID-19 datasets.

## VI. Conclusion

We have demonstrated that with current limited and challenging COVID-19 datasets, VGG19 model could be used to develop suitable deep learning-based tools for COVID-19 detection. The model is capable of classifying both Pneumonia vs Normal and COVID-19 vs Pneumonia conditions for multiple imaging modes including X-Ray, Ultrasound, and CT scan.

With very little data curation, we achieved considerable classification results using VGG19 from all imaging modes. Perhaps the most interesting observation is that the pre-trained models tuned very effectively for the Ultrasound image samples, which to the untrained eye appeared noisy and difficult to interpret. Both training curves and confusion matrix for both Ultrasound experiments are close to ideal. VGG19 also trained well against the X-Ray image corpus however, without modified thresholding we found that the proportion of false negatives was concerning but not unexpected given data quality challenges. Our finding that experiment 1A/2A yielded lower F1 scores and higher false negatives than experiments 1B/2B was unexpected since the manifestation of COVID-19 is itself a form of viral pneumonia. This may indicate that despite our attempts to remove sampling bias using N-CLAHE pre-processing there may still be systematic differences in the COVID-19 image data sets that leads the VGG19 classifier to more easily distinguish the COVID-19 images from the pneumonia images. A future research direction could be to isolate the lung field by segmentation for all image samples in order to remove noise and further reduce sampling bias. Our lower results against the CT image corpus were not surprising since the CT image slices available were not from a uniform patient location and displayed extremely high variability in both form and content.

Our study uncovers the challenging characteristics of the limited COVID-19 image datasets. This should be helpful for practitioners aiming to use these datasets for their research and development. We provided a pre-processing pipeline aimed to remove the sampling bias and improve image quality. Our preprocessed images are also made openly available for others to use. During our initial model selection experiment, we also found that both VGG16 and VGG19 classifiers provided good results within the experimental constraints of the small number of currently available COVID-19 medical images. While deeper networks generally struggled, they will perform better when larger datasets are available which will reduce the impact of data quality variation.

It is inevitable that the initial publicly available medical images for novel medical conditions such as COVID-19 will be low in number and poor in quality. In this situation we conclude that the VGG19 classifier with transfer learning provides a fast and simple to implement machine learning model for multiple imaging modes providing good results that may lead to clinically useful diagnostic tools.

Despite our promising results, we would urge great caution in the development of clinical diagnostic models using currently available COVID-19 image dataset. The effect of a false positive diagnosis of COVID-19 on an individual is the isolation of the individual and their contract traces and the mental anguish and stress caused by both the prognosis and the social isolation. A false positive COVID-19 diagnosis could result in an inappropriate course of treatment. The effects of a false negative COVID-19 diagnosis would also be devastating for the individual if that diagnosis led to an inappropriate lack of treatment, and also for the community since cautions against COVID-19 transmission may not be appropriately applied resulting in the further community spread of the disease.

As a higher quality corpus of COVID-19 diagnostic image data becomes available, it may be possible to produce clinically trusted deep learning-based models for the fast diagnosis of COVID-19 as distinguished from similar conditions such as pneumonia. Such a tool would prove invaluable in practice, where other diagnostic tests for COVID-19 are either unavailable or unreliable. As the COVID-19 spread progresses throughout remote and economically challenged locations, an ability to diagnose COVID-19 from a readily available and portable medical imaging equipment such as X-Ray and Ultrasound machines would help slow the spread of the disease and result in a better medical outcome for the population.

Data fusion concept allows us to combine multiple modes of data to improve model classification performance. Although data fusion comes with its own set of challenges [97], [98], it has been used successfully in other application areas such as remote sensing [99]–[101], action detection [102], and medical diagnosis and imaging [103], [104]. We plan to extend our study with multimodal data fusion when sufficient data is available.
